# Sweet syndrome successfully treated with upadacitinib

**DOI:** 10.1016/j.jdcr.2025.08.003

**Published:** 2025-08-19

**Authors:** Valeria Duque-Clavijo, Hung Q. Doan, John A. Das, Stephen K. Tyring

**Affiliations:** a Medical Student, Universidad de Los Andes, Bogotá, Colombia; b Division of Internal Medicine, Department of Dermatology, The University of Texas MD Anderson Cancer Center, Houston, Texas; c Division of Family Medicine, Department of Medicine, Western Reserve Hospital, Cuyahoga Falls, Ohio; d Department of Dermatology, The University of Texas Health Science Center at Houston, Houston, Texas

**Keywords:** JAK inhibitors, paraneoplastic syndrome, refractory neutrophilic dermatosis, Sweet syndrome, upadacitinib

## Introduction

Sweet syndrome, or acute febrile neutrophilic dermatosis, is a rare inflammatory condition. Histologically, it is defined by a dense neutrophilic infiltrate in the dermis without vasculitis. While the exact pathogenesis remains unclear, the condition is thought to involve dysregulated cytokine production and neutrophil activation, leading to inflammation.

It is classified into 3 subtypes: classical, malignancy-associated, and drug-induced. Systemic corticosteroids are first-line therapy, but prolonged use can cause adverse effects, necessitating alternatives. In refractory cases, second-line therapies such as colchicine and immunomodulators have been used with variable success.

Janus kinase (JAK) inhibitors have emerged as potential therapies for immune-mediated inflammatory disorders. Given the cytokine-driven nature of Sweet syndrome, JAK inhibitors like upadacitinib (Rinvoq) may provide an effective alternative for steroid-dependent or refractory cases. We present a case of chronic steroid-dependent Sweet syndrome successfully treated with upadacitinib, highlighting its potential in refractory cases.

## Case report

**Fig 1:**
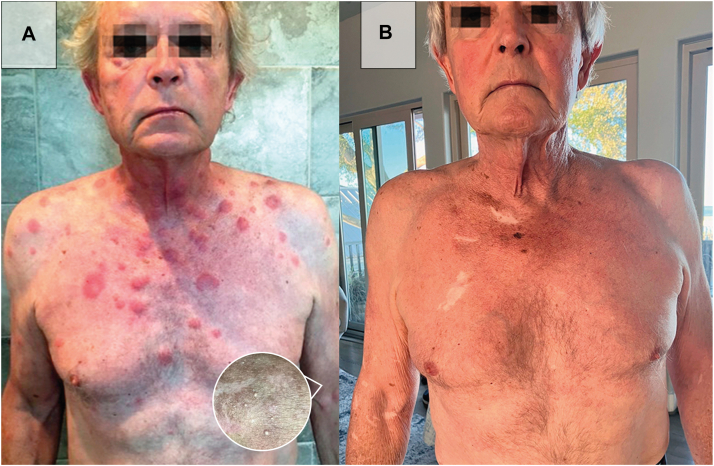
Anterior view of the patient. **A,** Exam findings on initial presentation of erythematous plaques and papules, some with central clearing. The lesions vary in size and appear to be nonuniform, with some coalescing into large patches. Zooming into his left arm, milia is present. **B,** Exam findings on the latest visit demonstrate complete clearance of his previous findings.

**Fig 2:**
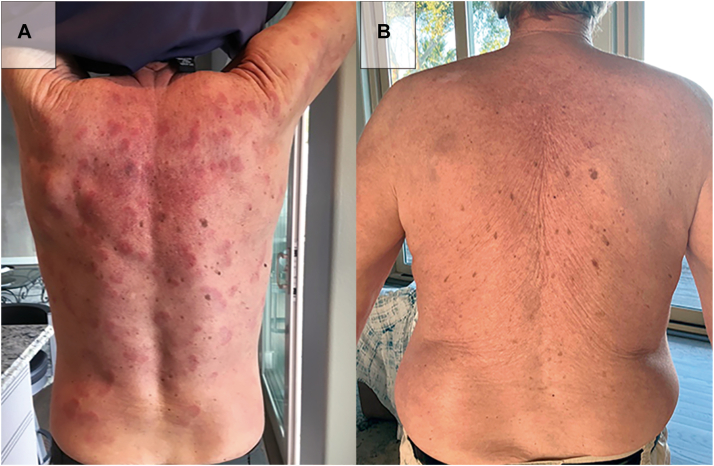
Posterior view of the patient. **A,** Exam findings on initial presentation of diffuse erythematous plaques and papules, some coalescing. There are areas of postinflammatory hyperpigmentation. **B,** Exam findings on the latest visit with complete clearance of his previous findings.

A 75-year-old male presented with a chronic, recurrent dermatosis that began in 2016 as a solitary, asymptomatic erythematous patch on the left chest. The lesion was neither pruritic nor painful. The patient intermittently experienced febrile episodes and myalgias. A skin biopsy confirmed the diagnosis of Sweet syndrome (Figs 1, *A* and 2, *A*).

The patient was initially treated with prednisone 20 mg daily, which effectively resolved his cutaneous lesions. However, attempts to taper the dose to 10 mg led to recurrence, necessitating a return to 20 mg daily. He remained on long-term corticosteroid therapy for 8 years. During this period, he developed corticosteroid-related adverse effects, including compressed vertebral discs, a sacral fracture requiring right hip replacement, and spondyloarthritis. Additionally, he developed milia on his upper extremities (Fig 1, *A*).

The patient was also diagnosed with stage III colorectal adenocarcinoma in September 2021 and underwent surgical resection followed by chemotherapy. He has remained cancer-free.

The patient first presented to our center in May 2023, when he was prescribed mycophenolate mofetil 250 mg twice daily for 1 month while continuing prednisone 20 mg daily. Two months later, as his skin lesions persisted without improvement, upadacitinib (Rinvoq) 15 mg daily was initiated while maintaining prednisone 20 mg daily.

One month after initiating upadacitinib, the dose was increased to 30 mg daily, and prednisone was tapered to 15 mg daily. After 7 months of treatment, the upadacitinib dose was further increased to 45 mg daily, enabling reduction of prednisone to 5 mg daily. By 12 months on upadacitinib, the signs and symptoms of Sweet syndrome, including the patient's erythematous plaques and papules, had cleared, and prednisone was discontinued (Figs 1, *B* and 2, *B*).

After 15 months on a stable dose of 45 mg daily without lesions, we attempted to taper upadacitinib to 30 mg daily. However, 1 month later, the skin lesions recurred, requiring a return to 45 mg daily. Since then, the patient's skin has remained clear.

## Discussion

Sweet syndrome, or acute febrile neutrophilic dermatosis, is a rare inflammatory condition marked by tender, erythematous papules, plaques, and nodules, often accompanied by fever and leukocytosis. Histologically, it shows a dense dermal neutrophilic infiltrate without vasculitis.^1^^,^^2^

Sweet syndrome has 3 categories:

• Classical Sweet syndrome typically affects women aged 30 to 50 years and may be preceded by an upper respiratory tract infection. Though more common in women, it has also been reported in men.^2^^,^^3^
• Malignancy-associated Sweet syndrome is often linked to hematologic malignancies, particularly acute myelogenous leukemia, and can serve as a paraneoplastic syndrome.^2^^,^^4^ The most frequently associated solid tumors are carcinomas, particularly those originating from the genitourinary system, breast, and gastrointestinal tract, with adenocarcinomas accounting for 57% of cases.^4^ In our case, the patient was diagnosed with colorectal adenocarcinoma 5 years after the onset of his initial skin lesion. It is also worth noting that resolution of the underlying malignancy may lead to improvement or resolution of the associated skin findings.
• Drug-induced Sweet syndrome is associated with medications such as nonsteroidal anti-inflammatory drugs, anticonvulsants, antipsychotics, and antineoplastics.^2^

The pathogenesis of Sweet syndrome likely involves cytokine dysregulation and neutrophil activation.^5^^,^^6^ Systemic corticosteroids remain first-line treatment and typically result in rapid symptom improvement. Additional treatment options include potassium iodide, colchicine, and second-line agents such as indomethacin, clofazimine, cyclosporine, and dapsone for refractory cases.^2^^,^^4^ One study reported the development of milia following the resolution of skin lesions in 3 patients treated with corticosteroids.^7^ Similarly, our patient developed milia on his arms between 2 flare-ups while on corticosteroid therapy.

Given an inadequate response to conventional therapies, upadacitinib, a JAK inhibitor, was started while tapering corticosteroids, with significant clinical improvement.

JAKs are intracellular enzymes that mediate signal transduction from cytokine and growth factor receptors, regulating processes from hematopoiesis to immune cell function. Within this signaling pathway, JAKs phosphorylate and activate signal transducers and activators of transcription, which regulate intracellular activity, including gene expression.^8^ JAK inhibitors, including upadacitinib, modulate this pathway by preventing the phosphorylation and activation of signal transducers and activators of transcription.

Upadacitinib is selective for JAK1 over other JAK isoforms, which is a key aspect of its mechanism of action. By preferentially inhibiting JAK1, upadacitinib disrupts the signaling pathways of pro-inflammatory cytokines, reducing inflammatory responses.^9^

Additionally, baricitinib, another JAK inhibitor, has been successfully used in treating refractory rheumatoid arthritis-associated Sweet syndrome,^10^ further supporting the potential role of JAK inhibitors in refractory Sweet syndrome. In this patient, upadacitinib was favored over baricitinib for its greater selectivity for JAK1, offering targeted immunomodulation with potentially fewer adverse effects in the setting of prior malignancy. This selectivity minimizes off-target inhibition of JAK2 and JAK3, which may lower the risk of cytopenias and other systemic complications.

However, JAK inhibitors carry risks, including a reported increased incidence of malignancies such as lymphomas and lung cancer. After discussing these risks, the patient opted to proceed.

Given the cytokine dysregulation and neutrophil activation characteristic of Sweet syndrome, JAK inhibitors may represent a promising off-label treatment. In our patient, an attempt to taper upadacitinib after 15 months on a stable dose of 45 mg daily was unsuccessful, indicating a possible need for long-term or indefinite therapy, with cautious future attempts at tapering based on ongoing clinical stability.

## Conflicts of interest

None disclosed.
